# LPLAT7 reutilizes unsaturated 1-lysophospholipids formed during lysosomal phospholipid degradation

**DOI:** 10.1016/j.jlr.2026.101064

**Published:** 2026-05-22

**Authors:** Yang Xu, Sujith Rajan, Colin K.L. Phoon, Mindong Ren, M. Mahmood Hussain, Michael Schlame

**Affiliations:** 1 Department of Anesthesiology, New York University Langone Medical Center, New York, NY, USA; 2 Department of Foundations of Medicine, New York University Long Island School of Medicine, Mineola, NY, USA; 3 Department of Pediatrics, New York University Langone Medical Center, New York, NY, USA

**Keywords:** lipidomics, lysophospholipid, phospholipase A, phospholipid/biosynthesis, phospholipid/metabolism

## Abstract

Lysosomal phospholipid degradation produces two types of metabolites, either 2-lysophospholipids with saturated fatty acids in sn-1 position or 1-lysophospholipids with unsaturated fatty acids in sn-2 position. They may either be degraded further or reused for phospholipid synthesis. We found that LPLAT7 (LPGAT1), an acyltransferase of the endoplasmic reticulum, reacylates specifically lysosome-derived 1-lysophospholipids that carry an unsaturated chain. The enzymatic activity of LPLAT7 was specific for stearoyl-CoA and 1-lyso-2-acyl positional isomers of unsaturated lysophospholipids. In Huh7 cells, *Lplat7* knockout prevented the reacylation of 1-lysophospholipids generated by the lysosomal degradation of exogenous ^2^H-phosphatidylcholine. Inhibition of lysosomal phospholipid degradation reduced the abundance of 1-stearoyl-2-unsaturated PC in Huh7 cells. *Lplat7* knockout blunted the loss of unsaturated lysophosphatidylcholine (LPC) in response to lysosomal inhibition, suggesting that LPLAT7 consumes unsaturated LPC formed by lysosomes. In mice, *Lplat7* knockout increased the concentration of unsaturated lysophospholipids, reduced the abundance of 1-stearoyl-2-unsaturated species of phosphatidylcholine, phosphatidylethanolamine, and phosphatidylserine, and inhibited the regeneration of cellular membranes. It also triggered the accumulation of triglycerides, confirming earlier reports that unsaturated lysophospholipids induce lipid droplet formation. Thus, by re-acylating unsaturated 1-lysophospholipids, LPLAT7 shifts lipid metabolism from the biogenesis of lipid droplets to the biogenesis of membranes.

Cells need to degrade phospholipids from endogenous and exogenous sources, such as autophagocytosed organelles and endocytosed lipoproteins. This takes place in lysosomes where two enzymes, PLBD2 and PLA2G15, have recently been identified that cleave fatty acids from both the sn-1 and sn-2 position, forming alternatively 1-lysophospholipids or 2-lysophospholipids (1). Since most phospholipids carry a saturated fatty acid at sn-1 position and an unsaturated fatty acid at sn-2 position, 1-lysophospholipids are typically unsaturated whereas 2-lysophospholipids are saturated. While either type of lysophospholipid may be degraded further, they may also be exported from lysosomes by SPNS1 (2, 3, 4). Once outside of lysosomes, lysophospholipids may become substrates of lysophospholipid acyltransferases (LPLATs) that convert them back into phospholipids by attaching coenzyme A-activated fatty acids (5). It has remained unclear though whether separate recycling pathways exist for 1-lysophospholipids and 2-lysophospholipids.

LPLATs constitute a family of 14 enzymes that have traditionally been named after the substrates they react with. This practice has caused confusion because substrate specificities may overlap between different LPLATs, and substrate specificities can be ambiguous. This dilemma prompted the introduction of a new nomenclature, in which each LPLAT is unequivocally assigned a number (5). However, despite decades of active research in the field, confusion has persisted about the function of some LPLATs.

Lysophosphatidylglycerol acyltransferase 1 (LPGAT1), renamed LPLAT7 (5), is a LPLAT located in the endoplasmic reticulum (ER) (6, 7). The function of LPLAT7 has remained highly controversial. The original name was given because in cell extracts expressing LPLAT7, lysophosphatidylglycerol (LPG) elicited a higher activity than other 1-acyl-2-lyso-phospholipids (6). However, 1-lyso-2-acyl-phospholipids were not tested in the study and data were confounded by high background activities. We have shown that LPLAT7, in contrast to most other LPLATs, prefers a free hydroxyl group at sn-1 rather than sn-2 position (8). Knockout of LPLAT7 decreased the stearate/palmitate ratio, which is consistent with sn-1 specific acyltransferase activity that is expected to affect saturated fatty acids (8).

The preference for 1-lyso-2-acyl-phospholipids has been confirmed by others (9, 10), but the exact head group specificity of LPLAT7 has remained uncertain. In mice, the ablation of *Lplat7* altered molecular species patterns of phosphatidylethanolamine (PE) and phosphatidylcholine (PC) (7, 8, 9, 10), suggesting lysophosphatidylethanolamine (LPE) and lysophosphatidylcholine (LPC) acyltransferase activity, but minor changes have also been reported in phosphatidylglycerol (PG) (7, 11). However, we did not find activity with LPC in vitro (8) whereas others contradicted this result (9). Usage of different LPC species confounded the interpretation. Thus, the published evidence suggests that LPLAT7 is a sn-1 specific acyltransferase with preference for saturated acyl-CoA’s but its preference for lysophospholipids with different head groups and acyl chains remains to be established.

Here we sought first, to clarify the native catalytic activity of LPLAT7 and second, to identify its biological function by determining the source of LPLAT7 substrates and the fate of LPLAT7 products. Our data suggest that LPLAT7 reacylates a wide spectrum of unsaturated 1-lyso-2-acyl-phospholipids with choline, ethanolamine, and serine head groups. Its purpose is to recycle unsaturated lysophospholipids generated by phospholipid degradation back into membrane phospholipids. The reutilization of unsaturated lysophospholipids is expected to make lipid metabolism more efficient by reducing the costs of lipid synthesis.

## Materials and Methods

### Materials

All lipids were purchased from Avanti Polar Lipids (https://www.avantiresearch.com). Chromatography columns were purchased from Restek (https://www.restek.com). Apolipoprotein B from human plasma (catalog number SRP6302) and oil red O (catalog number O9755) were obtained from Sigma-Aldrich (https://www.sigmaaldrich.com). Materials for quantitative Western blotting were obtained from LICOR (https://www.licor.com). Antibody raised against human LPLAT7 (LPGAT1) was purchased from Invitrogen (catalog number PA5-113143). All cell culture media were obtained from Gibco via Thermo Fisher Scientific (https://www.thermofisherscientific.com). Knockdown reagents were purchased from Origene (https://www.origene.com) and Gibco as specified. CRISPR/Cas9 genome editing was performed with reagents from Santa Cruz Biotechnology (https://www.scbt.com).

### Mice

All protocols were approved by the Institutional Animal Care and Use Committee of the NYU Grossman School of Medicine and conform to the Guide for the Care and Use of Laboratory Animals published by the National Institutes of Health. Mice were housed in a temperature-controlled facility under a 12-h light/dark cycle with free access to drinking water and food. The *Lplat7* (*Lpgat1*) knockout mouse strain (C57BL/6NJ-Lpgat1em1(IMPC)J/Mmjax, MMRRC stock #42167) was purchased from The Jackson Laboratory as a heterozygous breeding pair. Homozygous knockout mice were identified by standard genotyping of clipped tails. Mice were fed a standard chow diet and analyzed at the age of 3 months.

### Huh7 cells

Huh7 (human hepatocyte) cells were cultured in high-glucose DMEM supplemented with 10% fetal bovine serum, 100 IU/ml penicillin, and 100 μg/ml streptomycin (10% FBS-DMEM-PS). Cells were passaged when 90%–95% confluent. Stable knockout of *Lplat7* was created by CRISPR/Cas9 genome editing. After optimizing the plasmid concentration according to the manufacturer’s protocol, we added 3 μg of Lpgat1 double nickase plasmid (h) DNA (sc-411390-NIC, Santa Cruz) and 10 μl UltraCruz® transfection reagent (sc-395739, Santa Cruz) to 300 μl plasmid transfection medium (sc-108062, Santa Cruz) and placed the mixture on individual wells of 6-well plates. Huh7 cells (5 × 10^5^) were added to each well in 0.9 ml Opti-MEM medium and incubated for 8 h at 37°C in 5% CO_2_ and humidified atmosphere. Control cells were treated in parallel without plasmid. To screen for positive knockout clones, the cells were transfected and incubated in 10% FBS-DMEM-PS for 2 days followed by incubation in conditional medium supplemented with 2–3 μg/ml puromycin for 2–4 weeks until single colonies formed. The conditional medium was collected from the supernatant of cultured Huh7 cells after 1 day of culture and passed through a 0.2 μm filter. Puromycin was added to a final concentration of 2–3 μg/ml. We analyzed 126 clones by polymerase chain reaction of genomic DNA. Deletion clones were further analyzed by Western blots, which yielded 3 clones with confirmed ablation of LPLAT7. Of the 3, only one clone was routinely used in experiments because one clone showed poor viability and another clone became contaminated. As a result, replicate measurements were made in separate culture flasks of the same cell line.

### siRNA treatments

For *Lplat7* (*Lpgat1*) knockdown, 4 μl of 20 μm siRNA (*Lpgat1*-specific or scramble; SR322925B, OriGene) and 3 μl EndoFectin Max transfection reagent (EF013, https://www.thermofisherscientific.com/) were mixed in 200 μl Opti-MEM medium (11058-021, Gibco) and added to each well of a 6-well plate. Huh7 cells (2 × 10^5^) were added in 0.8 ml Opti-MEM medium and incubated for 8 h at 37°C in 5% CO_2_ and humidified atmosphere. Subsequently, the medium was changed to DMEM (Corning 10-013-CV) with 10% FBS-1xPenicillin-Streptomycin-L-Glutamine (Corning 30-009-CI) and incubation was continued for 2 days. Knockdown efficiencies were 90%–92% by quantitative RT-PCR and 42%–51% by Western blot analysis. For knockdown of *Plbd2* and *Pla2g15*, 3 μl EndoFectin Max and 4 μl siRNA (*Plbd2*- or Pla2g15-specific or scramble; Integrated DNA Technologies, https://www.idtdna.com) were added to 193 μl Opti-MEM to a final siRNA concentration of 10 nM. The mixture was incubated at 37°C for 10 min. An aliquot of 4 × 10^5^ exponentially growing Huh7 cells (WT or *Lplat7* knockout) were added to 6-well plates in 1 ml Opti-MEM serum-free medium. The transfected cells were incubated at 37°C in 5% CO_2_ and humidified atmosphere for 8 h and then changed to DMEM medium (Corning 10-013-CV) with 10% FBS-1xPenicillin-Streptomycin-L-Glutamine (Corning 30-009-CI). Cells were harvested after 2 days. Knockdown efficiencies were 95%–98% (*Pla2g15*) and 65%–70% (*Plbd2*) respectively when measured by quantitative RT-PCR.

### Subcellular membranes

Mouse liver tissue was homogenized in ice-cold isolation medium (0.25 M sucrose, 10 mM Tris, 1 mM EDTA, pH 7.4), using a glass-Teflon homogenizer, and spun at 1,000g for 5 min in a refrigerated centrifuge. The supernatant was centrifuged at 10,000g for 10 min. The supernatant of the 10,000g centrifugation was spun at 100,000g in a refrigerated ultracentrifuge, yielding ER membranes in the pellet. The pellet of the 10,000g centrifugation was resuspended in isolation medium and loaded onto a 30% Percoll medium as described (12). The density gradient was established by centrifugation at 95,000g for 30 min in a TLS55 swing-out rotor. The heavy fraction and the light fraction were collected and diluted in isolation medium (12). The heavy fraction was centrifuged at 10,000g to pellet mitochondria. The light fraction was centrifuged at 10,000g followed by centrifugation of the supernatant at 100,000g in order to pellet MAM (12).

### Lipidomics

Lipids were extracted from tissue samples and cell culture pellets. Each sample contained about 1–2 mg protein as determined by the Lowry method (13). Samples were suspended in methanol/chloroform (2:1) and incubated at 37°C for 30 min to denature proteins. At this point, two internal standard mixtures, including Mouse SPLASH and Cardiolipin Mix I were added (2–4 μl per mg protein) for absolute quantification. Chloroform and water were added, samples were vortexed, and phase separation was achieved by centrifugation. The lower phase was collected, dried under nitrogen, and re-dissolved in 0.1 ml chloroform/methanol (1:1). Lipids were analyzed by liquid chromatography electrospray-ionization tandem mass spectrometry (LC-ESI-MS/MS) using a QExactive HF-X instrument coupled directly to a Vanquish UHPLC (Thermo Fisher Scientific). An aliquot of 7 μl was injected into a Restek Ultra C18 reversed-phase column (100 × 2.1 mm; particle size 3 μm) that was kept at a temperature of 50°C. Chromatography was performed with solvents A and B at a flow rate of 0.15 ml/min. Solvent A contained 600 ml acetonitrile, 399 ml water, 1 ml formic acid, and 0.631g ammonium formate. Solvent B contained 900 ml 2-propanol, 99 ml acetonitrile, 1 ml formic acid, and 0.631g ammonium formate. The chromatographic run time was 40 min, changing the proportion of solvent B in a nonlinear gradient from 30% to 35% (0–2 min), from 35% to 67% (2–5 min), from 67% to 83% (5–8 min), from 83% to 91% (8–11 min), from 91% to 95% (11–14 min), from 95% to 97% (14–17 min), from 97% to 98% (17–20 min), from 98% to 100% (20–25 min), and from 100% to 30% (25–26 min). For the remainder of the run time the proportion of solvent B was kept at 30% (26–40 min). The mass spectrometer was operated in negative or positive ion mode and spectra were acquired in profile mode. The spray voltage was set to 4 kV and the capillary temperature was set to 350°C. MS1 scans were acquired at a resolution of 120,000, an AGC target of 1e6, a maximal injection time of 65 ms, and a scan range of 300–2000 m/z. MS2 scans were acquired at a resolution of 30,000, an AGC target of 3e6, a maximal injection time of 75 ms, a loop count of 13, and an isolation window of 1.7 m/z. The normalized collision energy was set to 30 and the dynamic exclusion time to 31 s. For lipid identification and quantitation, data were analyzed by the software LipidSearch 5.1.8 (Thermo Fisher Scientific). The general database was searched with a precursor tolerance of 2 ppm, a product tolerance of 0.01 Da, an intensity threshold of 1.0%, and an S/N threshold of 3. Glycerides were analyzed as [M + NH_4_] adducts in positive ion mode. PC was analyzed as [M + HCOO] adduct in negative ion mode and all other phospholipids as [M-H] ions in negative ion mode.

### Acyltransferase activity

LPLAT7 (LPGAT1) was expressed in *E*. *coli* as a fusion construct with maltose-binding protein (8). To assay LPLAT7 activity, we isolated E. coli membranes and incubated aliquots, containing 20 μg protein, in 1 ml Tris buffer (10 mM, pH 7.4) supplemented with 60 nmol stearoyl-CoA, and 60 nmol of various lysophospholipids. After 5 min of incubation at 37°C, the reaction was stopped by adding 2 ml methanol and 1 ml chloroform. Mouse SPLASH (5 μl) was added as internal standard, lipids were extracted and analyzed by LC-MS/MS as described above. Phospholipids formed by the enzyme were quantified by measuring peak areas of their MS1 ion currents at a mass tolerance of 2 ppm. Data were analyzed in the Quality Browser of Xcalibur 4.0 (Thermo Fisher Scientific). E-coli membranes expressing maltose-binding protein without LPLAT7 served as negative control. The LPLAT7 activity of Huh7 cell membranes, was assayed with 1-lyso-2-oleoyl-PE as acyl acceptor, using the same methodology as for E. coli membranes.

### Metabolism of deuterium-labeled PC

Huh7 cells were cultured in 10% FBS-DMEM for 12 h on 6-well plates at a density of 10^6^ cells per well. After that, cells were kept in 2% FBS-DMEM for another 12 h. The medium was removed and replaced by 1 ml labeling medium, containing either 100 μg 1-pentadecanoyl-2-D_7_oleoyl-PC (PC15:0_18:1D_7_) and 244 μg triolein (TG18:1_18:1_18:1) or 50 nmol (40 μg) 1-heptadecanoyl-2-dihomolinolenoyl-D_5_PC (D_5_PC17:0_20:3), 100 nmol (90 μg) triolein (TG18:1_18:1_18:1), and 30 μg apolipoprotein B. To prepare the medium, we dried lipids under a stream of nitrogen, added DMEM, and vortexed the solution and sonicated it in a water bath. If desired, apolipoprotein B was added at the end. Cells were harvested after different time periods and lipid extracts were analyzed by LC/MS as described above. Deuterium labeled precursors and their metabolites were quantified by measuring peak areas of their respective MS1 ion currents with a mass tolerance of 2 ppm. Data were analyzed in the Quality Browser of Xcalibur 4.0 (Thermo Fisher Scientific).

### ^13^C Labeling of murine hepatocytes

Primary hepatocytes were isolated from WT and knockout mice. Animals were anesthetized with isoflurane. The skin was disinfected with 70% ethanol and laparotomy was performed. A 22G cannula was inserted into the portal vein and the inferior vena cava was cut open. The liver was perfused through the portal vein with 50 ml prewarmed (37°C) Hank’s Balanced Salt Solution and with 50 ml Liver Digestion Medium containing collagenase (Gibco Cat. No. 17703-034). Liver tissue was carefully removed without gall bladder and placed into a sterile 50 ml tube containing 5 ml digestion medium. Liver tissue was dissected and gently agitated to release hepatocytes. An aliquot of 15 ml cold wash medium, containing low glucose DMEM, was added to the dissected liver and the preparation was filtered (70 μm) to remove debris and undigested tissue. Hepatocytes were collected by centrifugation and washed three times with 20 ml of cold wash medium. Washed hepatocytes were cultured in 10 ml low-glucose DMEM containing 10% FBS. Nonadherent cells were removed after 1 h at 37°C. Fresh medium was added and hepatocytes were cultured overnight. After that, the medium was replaced with ^13^C medium containing glucose-free DMEM, 2.5 g/L ^13^C_6_-glucose, and 10% FBS. The ^13^C medium was replaced every 24 h for up to 5 days. Cells were collected with a sterile cell scraper, spun at 200 g for 5 min, and kept at −80°C until analysis, which was performed as described (14). Briefly, lipids were extracted and injected into the LC-MS/MS system specified above. CL and PG species were identified by the software LipidSearch 5.1.8 (Thermo Fisher Scientific). Using Xcalibur 4.0, isotopomers of CL18:1_18:2_18:2_18:2, CL16:1_18:2_18:2_20:3, and PG16:0_18:1 were quantified in chromatographic windows of 0.2 min, centered around their peak retention times.

### Western blots

Quantitative Western blotting was performed with a commercial antibody raised against human LPLAT7 (LPGAT1). Total cell lysate (45 μg protein per sample) was loaded onto a 10% SDS-PAGE gel and developed under standard conditions with prestained protein markers. Proteins were transferred onto a PVDF membrane overnight. The PVDF membrane was treated with blocking buffer (927-70001, LI-COR) for 1 h and then with 2 μg/ml rabbit anti-hLPGAT1 polyclonal antibody (PA5-113143, Invitrogen) for 2 h. The membrane was washed 4 times with 0.1% Tween-20 in phosphate-buffered saline (PBS) followed by incubation with LiCor GAR-IDye800cw (926-32211, LI-COR) secondary antibody at a dilution of 1:10,000 for 1 h. The membrane was washed again 4 times with 0.1% Tween-20-PBS and rinsed twice with detergent-free PBS. Immunofluorescence imaging was performed with the LI-COR scanner.

### Oil red O staining

Cells were fixed with 4% paraformaldehyde-PBS and exposed to Oil red O working solution (0.3% Oil red O in isopropanol-water 6:4) for 10 min. Cells were imaged by a BioRad fluorescent imager in the bright field channel (gain 10, exposure time 345 ms, LED intensity 67).

### Statistical analysis

Bar graphs show mean values with standard deviation. The number of biological replicates varied from 3 to 8. Individual data are presented in most cases. If only means and standard deviations are given for the sake of visual clarity, the number of replicas is specified in the legend. Groups were compared by Student’s *t* test.

## Results

### LPLAT7 reacylates the sn-1 position of different unsaturated lysophospholipids

**Fig. 1:**
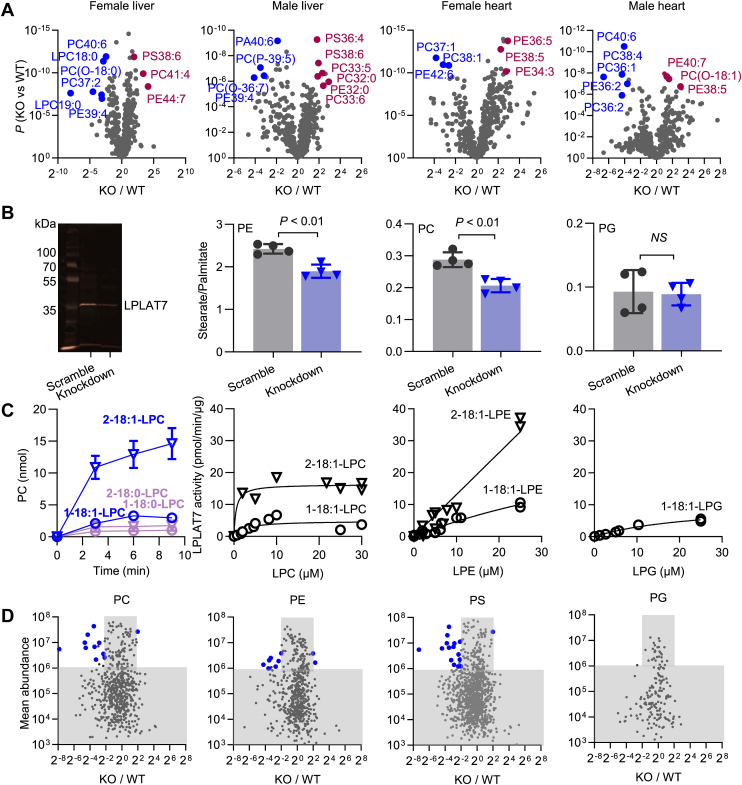
LPLAT7 reacylates the sn-1 position of unsaturated LPE and LPC. A, livers and hearts were harvested from 12 *Lplat7* knockout mice (6 females, 6 males) and 20 controls (12 females, 8 males). Their lipids were analyzed by LC-MS/MS. Volcano plots show species that decreased in knockouts in *blue* and species that increased in knockouts in *purple*. B, Huh7 cells were treated with siRNA targeting *Lplat7* or with scrambled siRNA. The expression of LPLAT7 was measured by Western blot with fluorescence antibodies. The stearate/palmitate ratios of PE, PC, and PG were measured by LC-MS/MS. C, acyl transferase activities were measured in *E*. *coli* membranes expressing LPLAT7 in the presence of stearoyl-CoA and different lysophospholipids containing stearoyl (18:0) or oleoyl (18:1) in sn-1 or sn-2 position (N = 3). D, scatter plots of molecular species of individual lipid classes were created from LC-MS/MS data collected in mouse heart and liver of male and female mice (12 *Lplat7* knockouts, 20 controls). Points in blue mark abundant species (peak area>10^6^) that change >4-fold. LPLAT, lysophospholipid acyltransferase; LPE, lysophosphatidylethanolamine; LPC, lysophosphatidylcholine; PE, phosphatidylethanolamine; PC, phosphatidylcholine; PG, phosphatidylglycerol.

To determine the native activity of LPLAT7, we first identified lipid species that changed their concentration in response to deletion of *Lplat7* from mice (Dataset 1). We had collected similar data for a previous paper (8) but here we studied a larger population, included both sexes, and analyzed heart and liver, two tissues that are functionally affected in the knockout (7, 11). Molecular species that experienced the largest change with the highest statistical significance, belonged almost entirely to the classes of PC and PE. In male liver, we also identified two phosphatidylserine (PS) species and one phosphatidic acid (PA) species, but PG species were not present among the high confidence responders (Fig. 1A). A systematic comparison of class-specific volcano plots confirmed that only PC, PE, and PS contained multiple molecular species that changed robustly in the LPLAT7 knockout, whereas PG, lyso-bis-phosphatidic acid, cardiolipin (CL), phosphatidylinositol, and PA did not (supplemental Fig. S1). The data suggest that LPLAT7 controls specifically the molecular compositions of PC, PE, and PS in vivo.

Second, we treated human hepatoma (Huh7) cells with small interfering RNA (siRNA) designed to suppress the *Lplat7* message. This treatment decreased the protein concentration of LPLAT7 and lowered the stearate/palmitate ratio of PE and PC but had no effect on the stearate/palmitate ratio of PG (Fig. 1B). These data are consistent with the effect of *Lplat7* knockout in mice and corroborate the previously reported stearoyl-CoA specificity of LPLAT7 (8).

Third, we expressed the enzyme in E. coli and measured acyltransferase activities with different lysophospholipids in LPLAT7-rich E. coli membranes. LPLAT7 reacted robustly with 1-lyso-2-oleoyl-PC (2-18:1-LPC) but not with 1-oleoyl-2-lyso-PC (1-18:1-LPC) or 1-lyso-2-stearoyl-PC (2-18:0-LPC) or 1-stearoyl-2-lyso-PC (1-18:0-LPC) (Fig. 1C). This reveals that LPLAT7 strongly prefers LPCs with a free hydroxyl group in sn-1 position and an unsaturated but not a saturated fatty acid in sn-2 position. We also compared the reaction rates of different oleoyl-containing lysophospholipid classes at variable substrate concentrations. LPE and LPC carrying the oleoyl residue in sn-2 position elicited much higher acyl transfer activities than LPE and LPC carrying the oleoyl residue in sn-1 position. The activity with 1-oleoyl-2-lyso-PG was as low as the activity with 1-oleoyl-2-lyso-PC and 1-oleoyl-2-lyso-PE (Fig. 1C). The data demonstrate strong specificity of LPLAT7 for unsaturated 1-lyso-2-acyl phospholipids and explain why we did not find any activity with 2-palmitoyl-LPC in our previous paper (8).

Fourth, we created scatter plots to determine the effect of LPLAT7 deletion on abundant species of different phospholipid classes. In PC, PE, and PS, we found several abundant species (peak area >10^6^) that changed their concentration more than 4-fold in response to *Lplat7* knockout. In contrast, none of the abundant species of PG experienced similar changes (Fig. 1D). This result corroborates that LPLAT7 re-acylates LPC, LPE, and lysophosphatidylserine (LPS) in vivo. The conclusion is consistent with the published head group specificity of LPLAT7 expressed in HEK293 cells, which shows the highest activity with LPC and LPE, some activity with LPS, and almost none with LPG, lysophosphatidylinositol, and lysophosphatidic acid (9).

In summary, we demonstrate strong specificity of LPLAT7 for unsaturated 1-lysophospholipids and stearoyl-CoA, which promotes the synthesis of 1-stearoyl-2-unsaturated species of PC, PE, and PS in vivo. The present results reconcile contradictory reports in the literature (6, 7, 8, 9). They show that LPLAT7 strongly prefers unsaturated over saturated lysophospholipids and 1-lysophospholipids over 2-lysophospholipids. They confirm that LPLAT7 is moderately specific for stearoyl-CoA (8, 9) and reacts with different lysophospholipid classes (LPC, LPE, LPS).

### Unsaturated lysophospholipids accumulate upon deletion of *Lplat7*

**Fig. 2:**
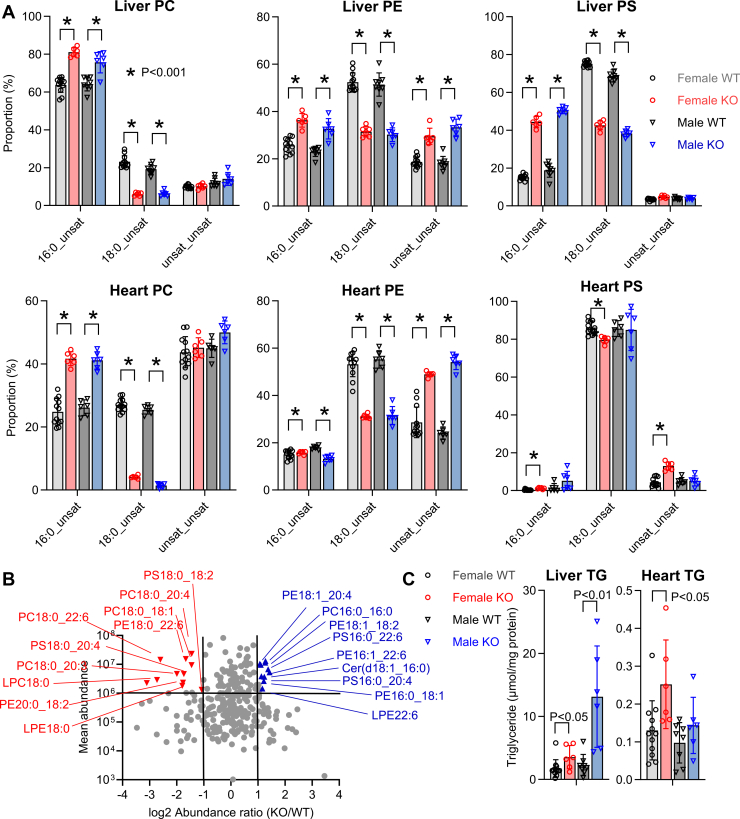
*Lplat7* knockout changes the composition of PC, PE, and PS and raises the level of triglycerides. Livers and hearts were harvested from *Lplat7* knockout mice (KO) and wild type (WT) to determine the abundance of lipid species by LC-MS/MS. A, *bar graphs* show the relative proportion of groups of PC, PE, and PS species. B, scatter plot shows molecular species of female livers. Abundant molecular species (peak area>10^6^) that change >2-fold are shown in *red* (decrease) and *blue* (increase) respectively. C, bar graphs show the concentration of triglycerides (TG). LPLAT, lysophospholipid acyltransferase; PC, phosphatidylcholine; PE, phosphatidylethanolamine; PS, phosphatidylserine.

Next we measured the species compositions of PC, PE, and PS in liver and heart. Knockouts contained less stearoyl-unsaturated species, which was compensated for by palmitoyl-unsaturated species (in PC and PS) or diunsaturated species (in PE) (Fig. 2A). A scatter plot of all hepatic lipids confirmed these compositional changes and the fact that they were confined to the lipid classes PC, PE, and PS (Fig. 2B). *Lplat7* knockout also increased the triglyceride content of liver and heart (Fig. 2C) but did not affect the molecular species composition of triglycerides (supplemental Fig. S2).

**Fig. 3:**
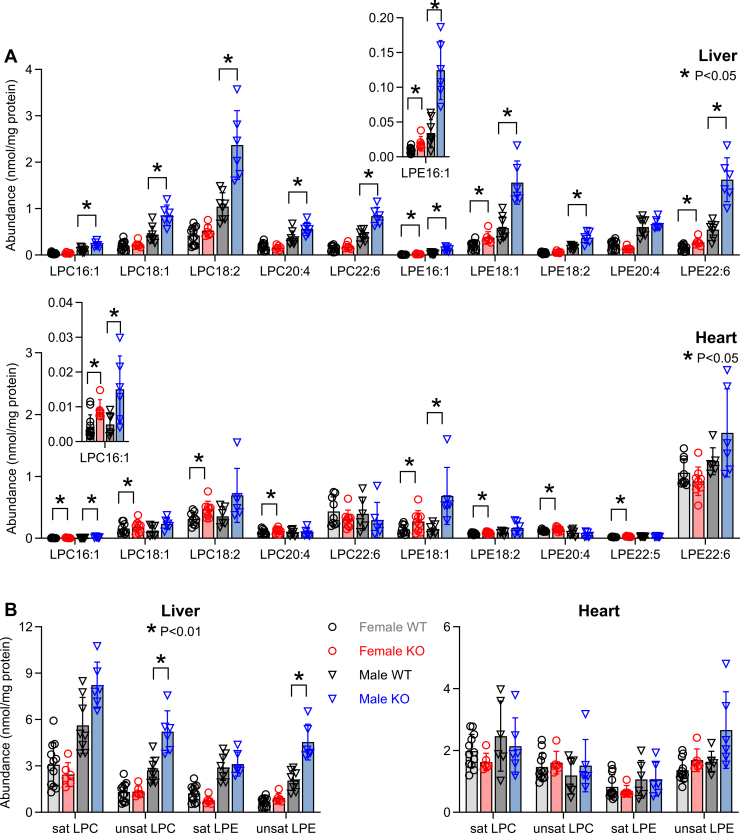
*Lplat7* knockout increases the abundance of unsaturated LPC and LPE. Livers and hearts were harvested from *Lplat7* knockout mice (KO) and wild type (WT) to determine the abundance of lysophospholipid species by LC-MS/MS. Bar graphs show the abundance of individual lysophospholipid species (A) or the abundance of groups of lysophospholipids (B). Sat, saturated; unsat, unsaturated. LPLAT, lysophospholipid acyltransferase; LPC, lysophosphatidylcholine; LPE, lysophosphatidylethanolamine.

Since 1-lyso-2-unsaturated-phospholipids are LPLAT7 substrates, we asked whether *Lplat7* knockout increases their concentration. Indeed, the concentration of most LPC and LPE species that carried unsaturated chains, were elevated in liver and heart of knockout mice (Fig. 3A). However, the total concentration of unsaturated LPC and LPE reached statistical significance only in male livers (Fig. 3B). It remains to be established why the hepatic lipidome of male mice responded stronger to *Lplat7* knockout than the hepatic lipidome of female mice both with regard to the increase in triglycerides and the increase in unsaturated lysophospholipids.

**Fig. 4:**
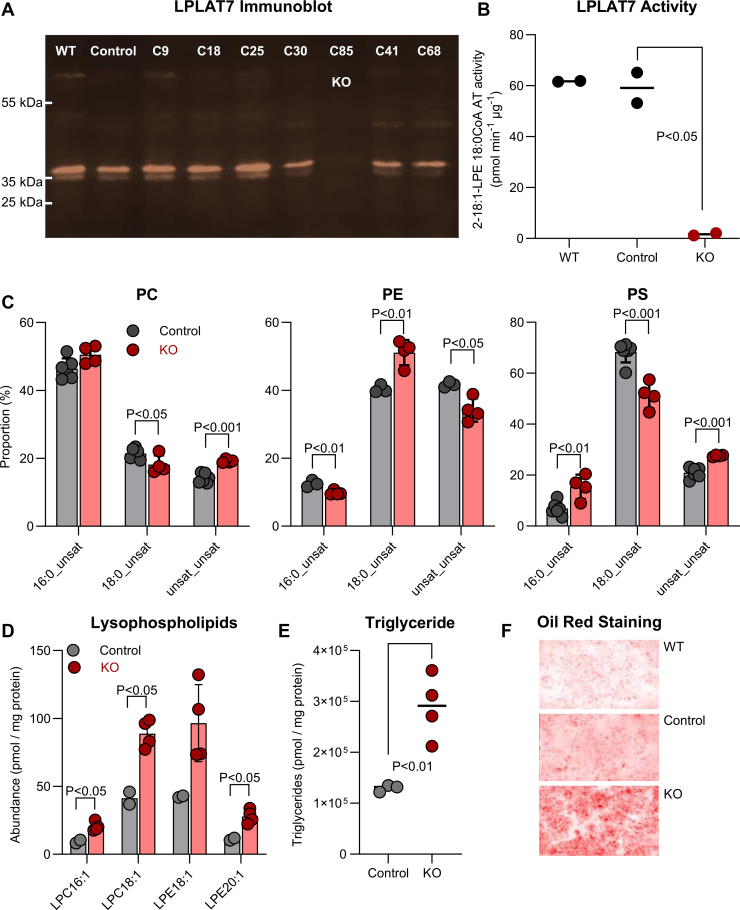
*Lplat7* knockout in Huh7 cells recapitulates the knockout phenotype of mice. *Lplat7* was deleted from Huh7 cells by CRISPR/Cas9 genome editing. A, clones were analyzed by immunoblotting with antibody against human LPLAT7. They included the Huh7 parent cell line (wild-type, WT), a clone without deletion (control), and several deletion clones, one of which exhibited knockout (KO) of *Lplat7*. B, LPLAT7 activity was measured by acyltransferase assay. C–E, the lipidomes of KO and control cells were analyzed by LC-MS/MS. F, the fat content of Huh7 cells was visualized by Oil *Red* O staining. Data are biological replicates. LPLAT, lysophospholipid acyltransferase.

To study LPLAT7 in human cell cultures, we created a Huh7 cell line with stable *Lplat7* knockout by CRISPR/Cas9 editing. Positive clones were selected by puromycin resistance and further tested by Western blots with antibody against human LPLAT7 (Fig. 4A). We identified a clone with LPLAT7 deletion, in which we confirmed the loss of LPLAT7 by acyltransferase assay with 1-lyso-2-oleoyl-PE and stearoyl-CoA (Fig. 4B). Because hepatocytes lack other enzymes to reacylate 1-lyso-2-oleoyl-PE, this assay is highly specific for LPLAT7 (8).

Huh7 cells with *Lplat7* deletion had lower proportions of 1-stearoyl-2-unsaturated species of PC and PS but surprisingly they contained a higher proportion of 1-stearoyl-2-unsaturated PE (Fig. 4C). The latter was almost entirely accounted for by an increase in PE18:0_20:3 at the expense of PE18:1_20:3. These species are abundant in Huh7 cells but not in mouse liver or other mouse tissues. We speculate that the increase in PE18:0_20:3 represents a unique effect of the knockout on cultured Huh7 cells. However, *Lplat7* knockout led to an increase of unsaturated LPC and LPE (Fig. 4D) and the accumulation of triglycerides (Fig. 4E) and lipid droplets (Fig. 4F), which is consistent with the data in *Lplat7* knockout mice.

In summary, *Lplat7* knockout (*i*) changed the species compositions of PC, PE, and PS but not of other phospholipids, (*ii*) increased the abundance of unsaturated lysophospholipids, most notably LPC and LPE, and (*iii*) increased the abundance of triglycerides in Huh7 cells and murine liver and heart.

### Substrates of LPLAT7 are formed by lysosomal phospholipid degradation

**Fig. 5:**
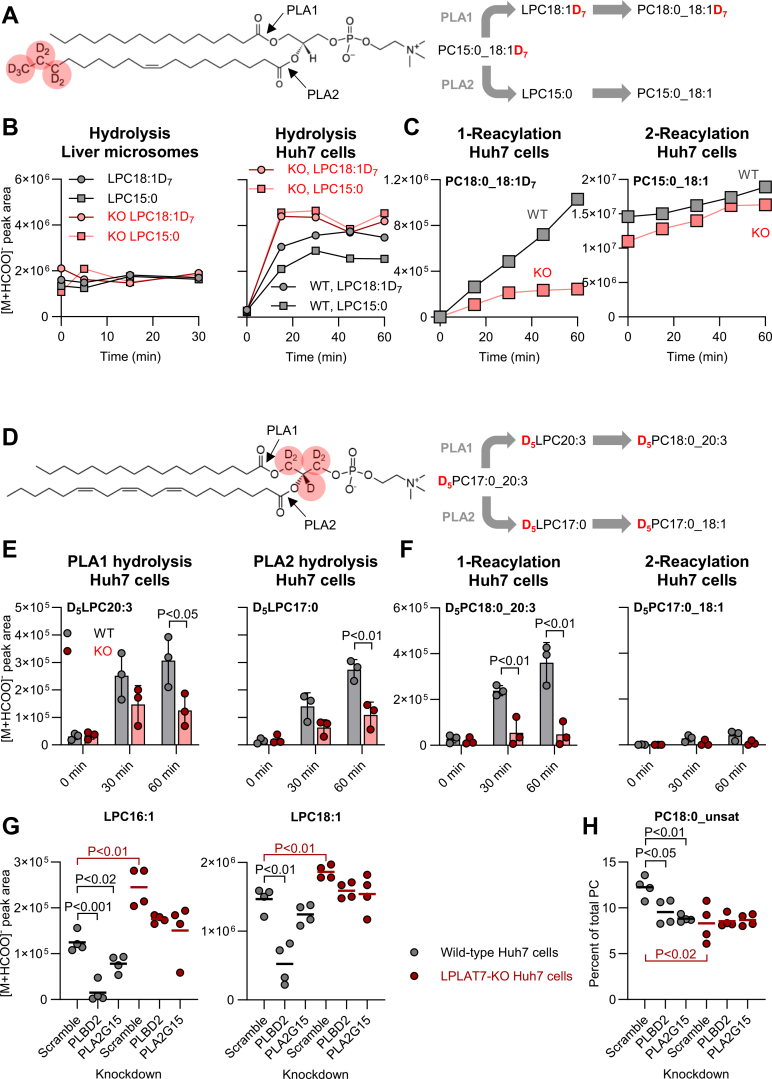
Lysosomal PC degradation delivers substrates to LPLAT7. A–C, Huh7 cells or microsomes from mouse liver, including wild-type (WT) and *Lplat7* knockout (KO), were incubated with lipid particles consisting of 1-pentadecanoyl-2-D_7_oleoyl-PC (PC15:0_18:1D_7_) and triglyceride (3:7 by weight). Lipid extracts were analyzed by LC-MS after different incubation times to measure metabolites of PC15:0_18:1D_7_. D–F, Huh7 cells (WT & KO) were incubated with artificial low-density lipoprotein consisting of 1-heptadecanoyl-2-dihomolinolenoyl-D_5_PC (D_5_PC17:0_20:3), triolein, and apolipoprotein B (24:56:20 by weight). Lipid extracts were analyzed by LC-MS after different incubation times to measure metabolites of D_5_PC17:0_20:3. G and H, Huh7 cells (wild-type and *Lplat7* knockout) were treated with siRNA targeting *Plbd2* or *Pla2g15* or with scrambled siRNA. PC and LPC were analyzed by LC-MS/MS. PC, phosphatidylcholine; LPLAT, lysophospholipid acyltransferase.

To identify the source of LPLAT7 substrates, we carried out tracer experiments with deuterium-labeled 1-pentadecanoyl-2-oleoyl-PC (PC15:0_18:1D_7_). Hydrolysis of PC15:0_18:1D_7_ by phospholipase A1 (PLA1) or A2 (PLA2) yields specific isotope-labeled or odd-chain metabolites that are detectable by mass spectrometry (Fig. 5A). First, we checked whether those metabolites can be generated directly in the ER where LPLAT7 is located, but we were unable to observe any products of PLA1 or PLA2 hydrolysis when we incubated PC15:0_18:1D_7_ with liver microsomes from WT or knockout mice. In contrast, such products became readily detectable when PC15:0_18:1D_7_ was supplied as lipid particles to Huh7 cells. Lipid particles are known to enter cells by endocytosis after which they are transferred to lysosomes for degradation (15, 16). When we added PC15:0_18:1D_7_ to Huh7 cells, both the PLA1 product (LPC18:1D_7_) and the PLA2 product (LPC15:0) were formed, reaching steady state within 30 min. This was well before PC15:0_18:1D_7_ was depleted, suggesting that the new LPCs were consumed by secondary metabolic reactions (Fig. 5B). Indeed, we measured a steady increase in the signal intensities of 1-stearoyl-2-D_7_oleoyl-PC (PC18:0_18:1D_7_) and 1-pentadecanoyl-2-oleoyl-PC (PC15:0_18:1), two species formed by reacylation of the respective LPCs. Importantly, the formation of PC18:0_18:1D_7_, but not the formation of PC15:0_18:1, was inhibited by *Lplat7* knockout. (Fig. 5C). These data demonstrate that exogenous 1-pentadecanoyl-2-D_7_oleoyl-PC is readily hydrolyzed by PLA1 and PLA2 and that LPLAT7 specifically reacylates PLA1-generated 1-lyso-2-D_7_oleoyl-PC. Since endocytosed lipids are metabolized in lysosomes, our data indicate that lysosomal pathways supply substrates to LPLAT7.

To further investigate the source of LPLAT7 substrates, we added a different precursor (1-heptadecanoyl-2-dihomolinolenoyl-D_5_PC, D_5_PC17:0_20:3) that carried deuterium atoms in the glycerol group. This gives rise to another set of deuterium-labeled metabolites in response to PLA1 and PLA2 hydrolysis (Fig. 5D). To better account for the native state of extracellular lipids, we complexed the precursor with artificial low-density lipoprotein (apolipoprotein B + triolein + D_5_PC17:0_20:3, 20:56:24 by weight) before adding it to Huh7 cells. D_5_PC17:0_20:3 was taken up within 30 min (supplemental Fig. S3) and hydrolyzed by PLA1 and PLA2, forming D_5_LPC20:3 and D_5_LPC17:0 respectively (Fig. 5E). The PLA1 product D_5_LPC20:3 was reacylated to D_5_PC18:0_20:3 and to D_5_PC16:0_20:3 but the PLA2 product D_5_LPC17:0 was not reacylated to any measurable extent. The reacylation of D_5_LPC20:3 to D_5_PC18:0_20:3 was strongly inhibited in knockout cells demonstrating that it was catalyzed by LPLAT7. Knockout had also some effect on the reacylation to D_5_PC16:0_20:3, suggesting some activity of LPLAT7 with palmitoyl-CoA (Fig. 5F and supplemental Fig. S3). Together the tracer experiments demonstrate that LPLAT7 reacylates unsaturated lysophospholipids derived from endocytosed phospholipids, implicating lysosomes in providing substrates to LPLAT7.

To directly test the involvement of lysosomal phospholipid degradation, we inhibited the lysosomal phospholipases PLBD2 and PLA2G15 by siRNA treatment of Huh7 cells. Knockdown of PLBD2 and PLA2G15 reduced the concentrations of the two major LPC species (LPC16:1 and LPC18:1), indicating inhibition of PC recycling through the lysosomal pathway. Knockout of *Lplat7* blunted the effect of lysosomal inhibition on LPC concentrations consistent with the idea that LPLAT7 consumes unsaturated LPCs (Fig. 5G). Importantly, lysosomal inhibition reduced the proportion of 1-stearoyl-2-unsaturated species in PC to the same extent as knockout of *Lplat7*, suggesting that both lysosomal degradation of PC and LPLAT7 activity are necessary to generate 1-stearoyl-2-unsaturated PCs (Fig. 5H). These data confirm that lysosomal phospholipid degradation provides substrates to LPLAT7.

In summary, we have shown that PC recycling by lysosomes forms 1-lyso-2-unsaturated-PC and 1-saturated-2-lyso-PC. Of those two LPC species, only 1-lyso-2-unsaturated-PC is reacylated by LPLAT7. Our data suggest that lysosomal phospholipid degradation is the principal source of LPLAT7 substrates.

### Products of LPLAT7 are exported from the ER

**Fig. 6:**
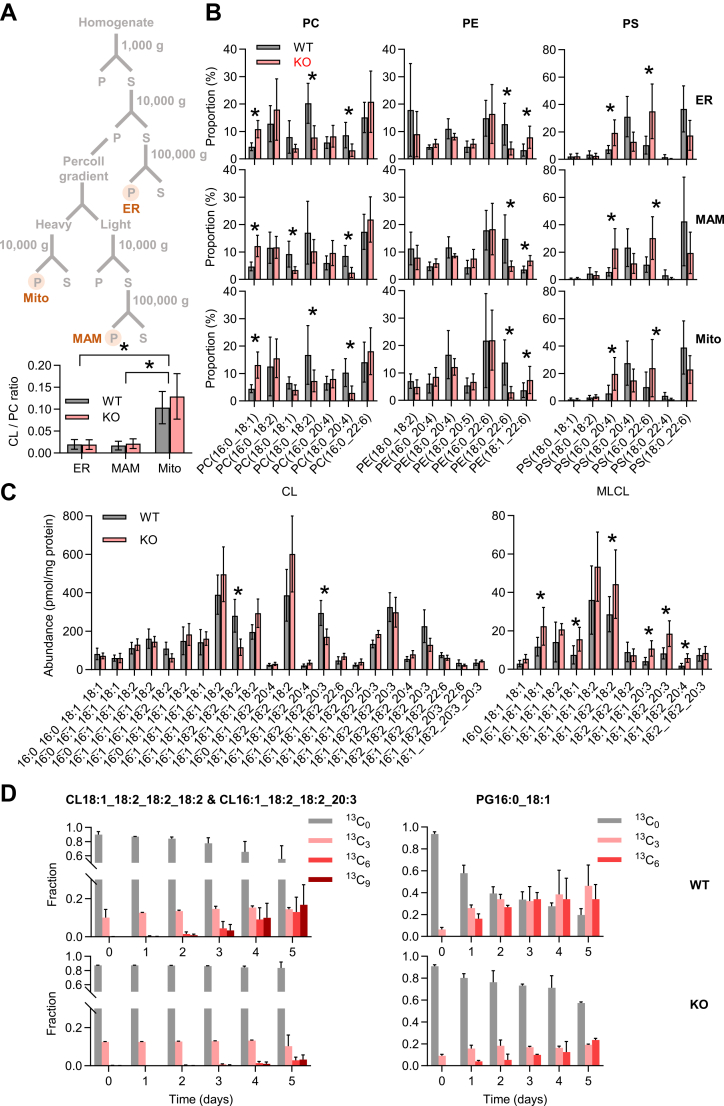
LPLAT7 products are exported to mitochondria. A and B, ER, MAM, and mitochondria (Mito) were isolated from mouse liver homogenates by a combination of differential centrifugation and Percoll gradient centrifugation (P, pellet; S, supernatant). Lipids of the subcellular fractions were analyzed by LC-MS/MS (N = 4). The 7 most abundant species of PC, PE, and PS are shown. C, livers were harvested to determine the abundance of CL and MLCL species by LC-MS/MS (N = 6). D, hepatocytes were isolated from mouse liver and cultured in the presence of ^13^C_6_-glucose. Cells were harvested after different incubation periods and analyzed by LC-MS to determine the distribution of isotopomers in CL and PG species (N = 3). Asterisks indicate statistically significant differences between wild-type (WT) and *Lplat7* knockout (KO) (*P* < 0.05). CL, cardiolipin; ER, endoplasmic reticulum; LPLAT, lysophospholipid acyltransferase; MLCL, monolyso-cardiolipin; PG, phosphatidylglycerol.

LPLAT7 has been shown to reside in the ER and specifically in the ER regions tethered to mitochondria (6, 7). To determine whether the products of LPLAT7 are confined to the ER or are transferred to mitochondria, we isolated ER, mitochondria, and mitochondria-associated membranes (MAM) from liver homogenate. We confirmed that only mitochondria but not ER and MAM, contained CL, an established mitochondrial marker (Fig. 6A). *Lplat7* knockout changed the species composition of PC, PE, and PS in all 3 compartments (Fig. 6B), demonstrating that phospholipids formed by LPLAT7 in the ER are exported to mitochondria and potentially to other cellular membranes.

Deletion of *Lplat7* in mice did not change the abundance of CL in liver (3.7 ± 0.5 vs. 3.8 ± 0.9 nmol/mg protein) but increased the concentrations of monolyso-cardiolipin (MLCL) (Fig. 6C). As a result, the MLCL/CL ratio increased from 0.022 ± 0.007 to 0.055 ± 0.020 in liver (N = 6, *P* = 0.0002) and from 0.012 ± 0.005 to 0.048 ± 0.009 in heart (N = 6, *P* = 0.001). The rise of MLCL is a rare pathologic finding characteristic of Barth syndrome (17) where it is caused by accelerated CL degradation (18, 19). Although the increase in the MLCL/CL ratio was small compared to Barth syndrome, we asked whether CL metabolism was also increased in *Lplat7* knockouts. To answer the question, we measured the incorporation of ^13^C isotopes into the glycerol moieties of CL and its precursor in isolated hepatocytes of *Lplat7* knockout mice and their controls. Deletion of *Lplat7* drastically reduced the rate of ^13^C incorporation into CL and PG (Fig. 6D), indicating reduced de novo synthesis of CL. Thus, the cause of MLCL accumulation in *Lplat7* knockout mice is different from the cause in Barth syndrome. While the pathological mechanism remains to be established, reduced CL synthesis and reduced synthesis of other lipids, as shown before (8), are consistent with global impairment of membrane regeneration in *Lplat7* knockout mice.

In summary, we have shown that LPLAT7 contributes phospholipids to mitochondria and that the deletion of *Lplat7* may limit the capacity of hepatocytes to regenerate mitochondrial membranes. The data are consistent with diminished oxidative capacity of liver mitochondria in *Lplat7* knockout mice (7) and with the presence of cardiomyopathy (11), which is often triggered by mitochondrial dysfunction.

## Discussion

We have demonstrated that LPLAT7 reacylates unsaturated 1-lysophospholipids formed by lysosomal phospholipid degradation. The enzyme has strong specificity for 1-lyso-2-unsaturated phospholipids and produces mostly 1-stearoyl-2-unsaturated species of PC, PE, and PS in vivo. We found these products not only in the ER, where LPLAT7 is located, but also in mitochondria and in MAM. We speculate that LPLAT7 was misidentified as LPGAT1 (6) because LPG, having 3 unesterified hydroxyl groups, is prone to intramolecular acyl migration. As a result, 1-lyso-PG may become available when 2-lyso-PG is used as substrate. However, our lipidomics data do not demonstrate any involvement of LPLAT7 with PG in vivo.

**Fig. 7:**
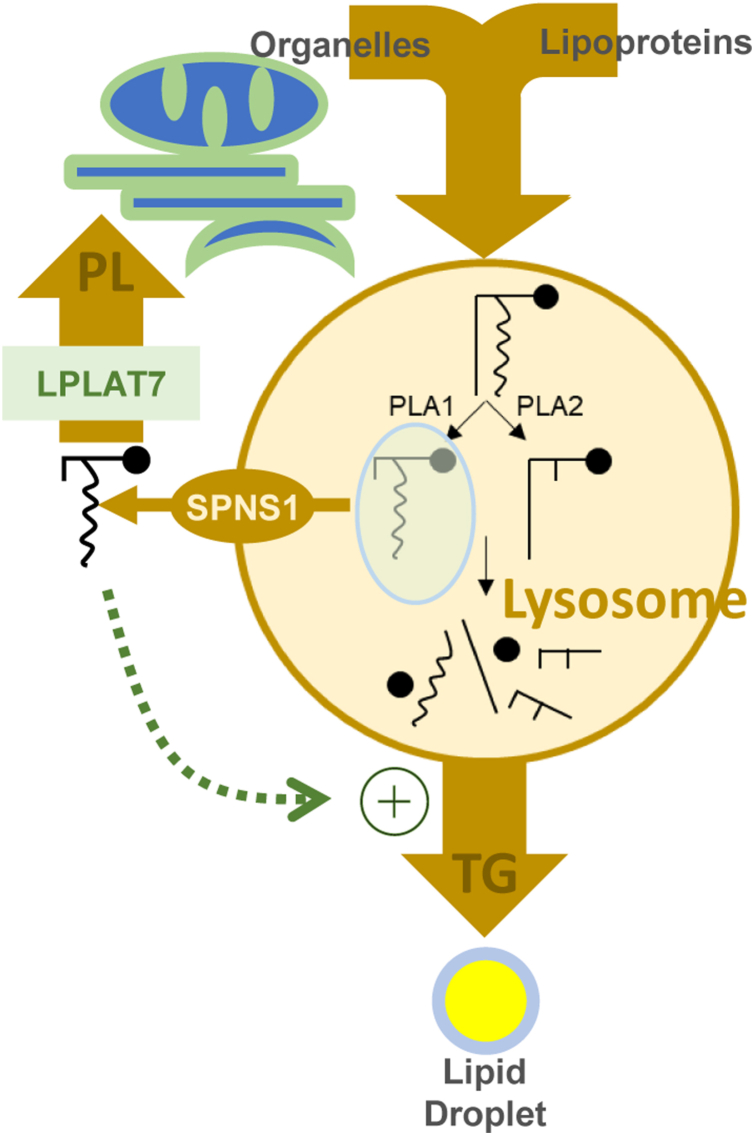
PLA1-SPNS1-LPLAT7 pathway recycles unsaturated 1-lysophospholipids into phospholipids. Phospholipids (PL) are hydrolyzed in lysosomes by PLA1 and PLA2 activities, forming unsaturated 1-lysophospholipids and saturated 2-lysophospholipids. Lysophospholipids are either degraded or reused for PL synthesis. Reutilization of unsaturated 1-lysophospholipids requires the lysosomal transporter SPNS1 and the acyltransferase LPLAT7. Lack of LPLAT7 causes a rise in unsaturated 1-lysophospholipids, which triggers the synthesis of triglycerides (TG) for fat storage. LPLAT, lysophospholipid acyltransferase; PLA, phospholipase A.

Lysosomes break down phospholipids by stepwise removal of fatty acids, which yields first a lysophospholipid and then a glycerophosphodiester. Either metabolite can be exported from lysosomes with the help of specific carriers. Lysophospholipids, in particular, are transferred across the lysosomal membrane by SPNS1 (2, 3, 4). Deficiency of SPNS1 leads to accumulation of lysophospholipids within lysosomes, which causes a lysosomal storage disease (2) presenting with neurologic and hepatic dysfunction (4). Our results suggest that LPLAT7 is a critical enzyme operating downstream of SPNS1 (Fig. 7). We show that LPLAT7 reacylates specifically 1-lysophospholipids that carry unsaturated fatty acids in sn-2 position. Unsaturated lysophospholipids are particularly valuable for the resynthesis of phospholipids because unsaturated fatty acids are less ubiquitous in food and more costly to synthesize than saturated fatty acids. Therefore, it is not surprising that cells have developed a robust mechanism to salvage unsaturated lysophospholipids.

Our data suggest that the PLA1-SPNS1-LPLAT7 salvage pathway is important for the regeneration of cellular membranes. We have shown that LPLAT7 contributes phospholipids to mitochondria and that lack of LPLAT7 leads to the accumulation of damaged mitochondria in primary hepatocytes. Others have shown that *Lplat7* knockout causes mitochondrial dysfunction in mouse liver (7, 11). We do not know whether LPLAT7 supplies phospholipids also to other cellular compartments but the large changes in the lipidome caused by *Lplat7* knockout, suggest a substantial involvement of the enzyme in the total phospholipid supply. Consistent with that, the phenotype of *Lplat7* deletion manifests as low birth weight, hepatic steatosis, cardiomyopathy, and premature death (7, 8, 11).

A growing body of evidence has implicated LPLAT7 (previously called LPGAT1) in the regulation of lipid metabolism. Expression of LPLAT7 is controlled by the same micro-RNA that influences hepatic lipid synthesis and lipoprotein assembly (20). Ablation of LPLAT7 in mice causes fatty liver (7, 21) and *Lplat7* variants are associated with obesity (22). These data hint at an undetermined role of LPLAT7 in rewiring lipid fluxes. Here we have shown that *Lplat7* deletion causes a rise in unsaturated lysophospholipids and others have shown that unsaturated lysophospholipids downregulate de novo lipogenesis and upregulate lipid droplet formation via transcriptional regulation (23, 24). Our data corroborate the accumulation of lipid droplets in response to increased levels of unsaturated lysophospholipids. We therefore propose a regulatory function of LPLAT7 in the redistribution of cellular lipid fluxes (Fig. 7). Low LPLAT7 activity prevents phospholipid resynthesis and causes high 1-lysophospholipid levels, which stimulates triglyceride synthesis for the purpose of fat storage. Conversely, high LPLAT7 activity drives phospholipid resynthesis and depletes the 1-lysophospholipid pool, which decreases fat storage. LPLAT7 may act in conjunction with other regulators, such as mTORC1 that has also been shown to balance lipogenesis against fat storage via a lysosome-dependent mechanism (25).

In conclusion, LPLAT7 rescues unsaturated 1-lysophospholipids by recycling them back into phospholipid synthesis. This pathway salvages a set of valuable metabolites that would otherwise have to be resynthesized at high metabolic expense. Our data do not support any significant involvement of LPLAT7 in the remodeling of PG, a mitochondrial lipid, but instead suggest that mitochondrial dysfunction may result from a defect in phospholipid regeneration in *Lplat7* knockouts. Finally, LPLAT7 is a metabolic regulator that balances triglyceride synthesis against phospholipid synthesis.

## Data availability

All data are contained within the article.

## Supplemental data

This article contains supplemental data.

## Conflict of interest

The authors declare that they have no conflicts of interest with the contents of this article.
