# Supplementary material for: LPLAT7 reutilizes unsaturated 1-lysophospholipids formed during lysosomal phospholipid degradation
Source: J Lipid Res. 2026 May 22;67(6):101064. doi: 10.1016/j.jlr.2026.101064 (PMC13277423; doi:10.1016/j.jlr.2026.101064)
Supplement: Supplemental Fig. S1 [file mmc1.pdf]

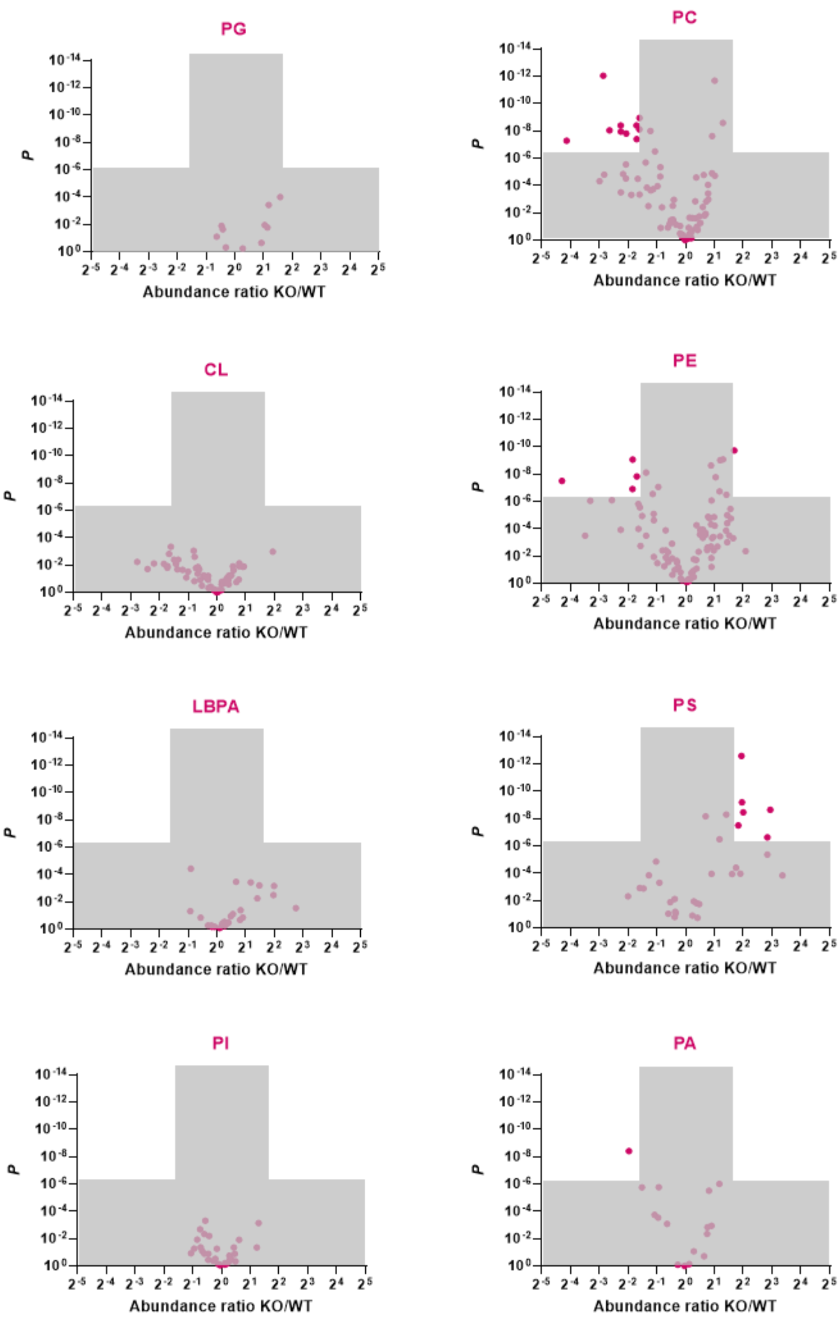

Figure S1. *Lplat7* knockout changes the composition of PC, PE, and PS.

Livers were harvested from 12 *Lplat7* knockout mice (6 females, 6 males) and 20 controls (12 females, 8 males). The molecular species compositions of lipid classes were determined by LC-MS/MS. Volcano plots show molecular species of the indicated phospholipid class. Molecular species that respond to *Lplat7* knockout with the largest change (>2.5-fold) and the highest statistical significance ($P<10^{-6}$) are located outside the shaded zone.
