# Supplementary material for: LPLAT7 reutilizes unsaturated 1-lysophospholipids formed during lysosomal phospholipid degradation
Source: J Lipid Res. 2026 May 22;67(6):101064. doi: 10.1016/j.jlr.2026.101064 (PMC13277423; doi:10.1016/j.jlr.2026.101064)
Supplement: Supplemental Fig. S2 [file mmc2.pdf]

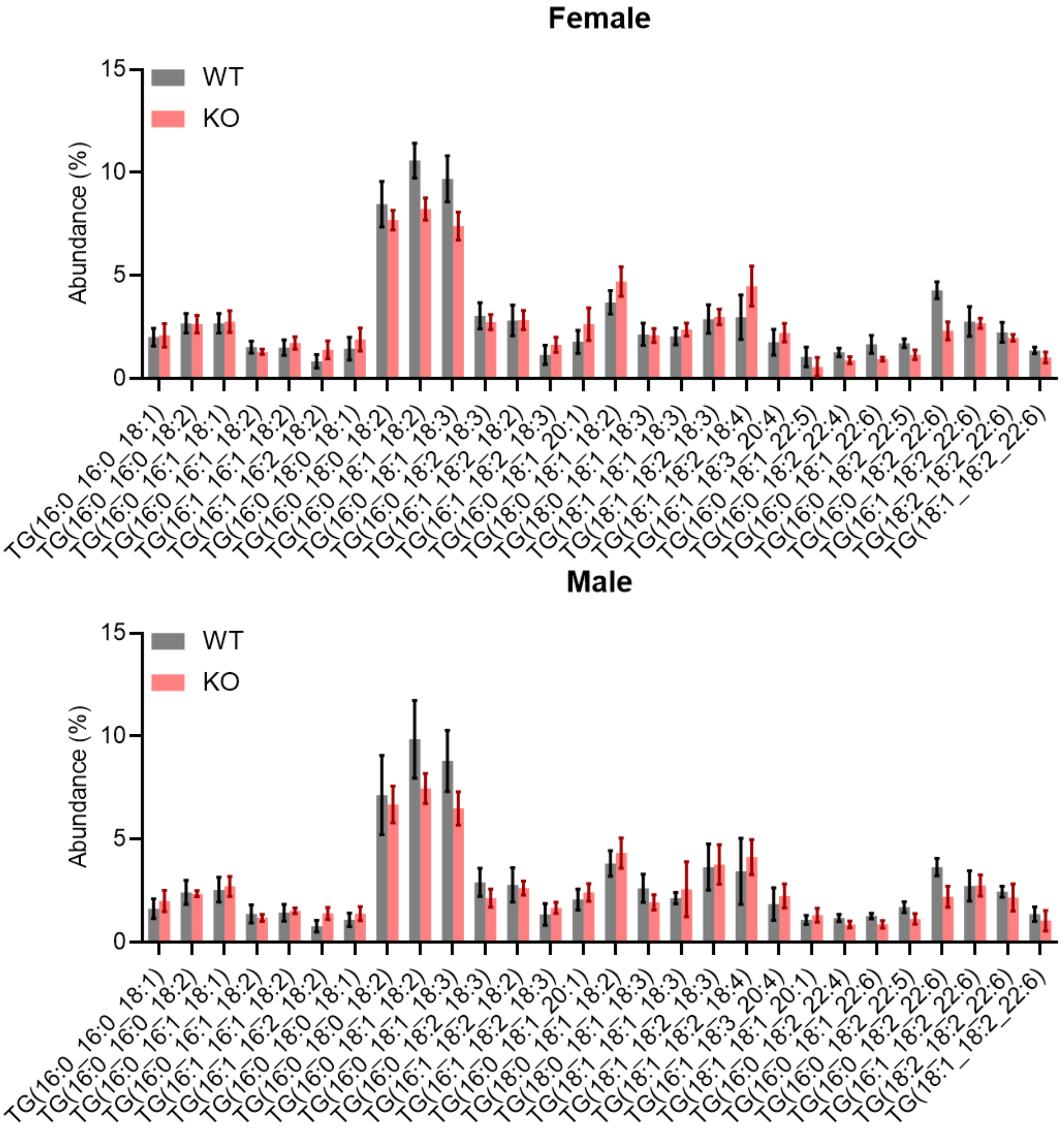

Figure S2. *Lplat7* knockout does not change the molecular species composition of triglycerides.

Livers were harvested from *Lplat7* knockout (KO) mice and wild type (WT). The molecular composition of triglycerides was measured by LC-MS/MS. Only species that contribute >1% of total TG are shown. Data are means with standard deviations of 6 replicas.
