# Supplementary material for: LPLAT7 reutilizes unsaturated 1-lysophospholipids formed during lysosomal phospholipid degradation
Source: J Lipid Res. 2026 May 22;67(6):101064. doi: 10.1016/j.jlr.2026.101064 (PMC13277423; doi:10.1016/j.jlr.2026.101064)
Supplement: Supplemental Fig. S3 [file mmc3.pdf]

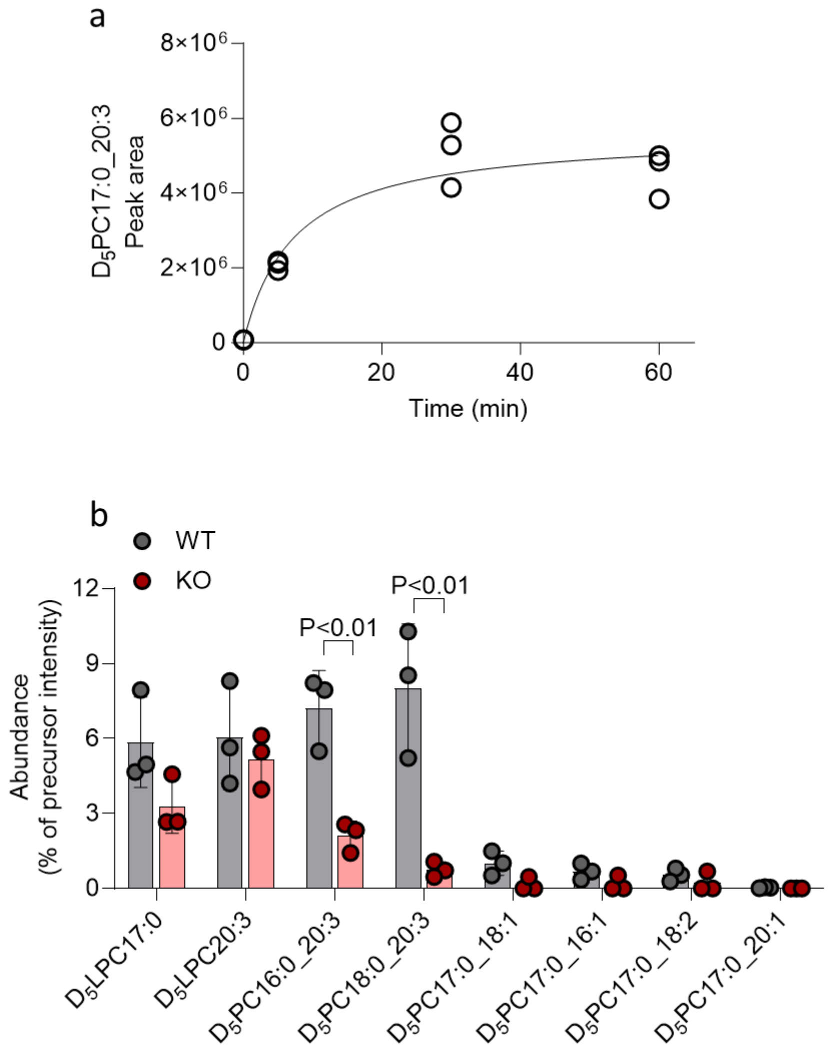

Figure S3. Huh7 cells take up deuterium-labeled PC, hydrolyze it by PLA1 and PLA2, and reacylate the PLA1-derived LPC.

Huh7 cells, including wild-type (WT) and *Lplat7* knockout (KO), were incubated with artificial low-density lipoprotein consisting of 1-heptadecanoyl-2-dihomolinolenoyl-D_5PC (D_5PC17:0_20:3), triolein, and apolipoprotein B (24:56:20 by weight). Lipid extracts were analyzed by LC-MS at different incubation times. **a**, The amount of cell-associated D_5PC17:0_20:3 increased over time. **b**, Deuterium labeled LPCs (D_5LPC17:0, D5LPC20:3) and their reacylation products were measured after 60 minutes.
